# A Johari-Inspired Framework for Population Gap Analysis: Linking Underdiagnosis, Overdiagnosis, Diagnostic Competition, and Prevention

**DOI:** 10.7759/cureus.110769

**Published:** 2026-06-13

**Authors:** Sandeep Das, Debabrata Tripathy, Ponnarasu Ponnu

**Affiliations:** 1 Department of Community Medicine and Family Medicine, All India Institute of Medical Sciences, Bhubaneswar, Bhubaneswar, IND

**Keywords:** care cascade, diagnostic competition, diagnostic performance, gap analysis, health system research, johari window, population health management, population modeling

## Abstract

Linear care cascades (the Rule of Halves and awareness-treatment-control frameworks) quantify attrition among system-recognized cases but cannot represent true disease that remains undiagnosed, system-labeled disease that is absent or non-actionable, or symptomatic individuals whose diagnostic encounters are consumed by competing labels. A structural solution to these simultaneous limitations does not exist within current population health methodology.

We conducted a narrative review of prior healthcare uses of the Johari Window, empirical applications and limitations of linear gap frameworks, evidence on the burden and consequences of underdiagnosis and inappropriate labeling, and literature on diagnostic competition and competing-diagnosis bias. We searched PubMed/Medical Literature Analysis and Retrieval System Online (MEDLINE) and Google Scholar (2000 to January 2026) using terms spanning Johari Window applications in health, population care cascades, underdiagnosis, overdiagnosis, and competing diagnoses. We adapted the Johari Window by jointly classifying populations along two operational axes, true disease state and health-system labeling state, and applied a priority-ordered classification rule to yield four mutually exclusive, jointly exhaustive quadrants.

The adapted framework generates four quadrants: Open (disease present, target-disease label present); Hidden (disease present, no label); Facade (label present without actionable target diagnosis, with two mechanistically distinct subtypes: type I diagnostic misclassification and type II diagnostic competition); and Unknown (disease absent, no label, stratified into at-risk and not at-risk). Four system-level diagnostic performance metrics (system sensitivity, system specificity, positive predictive value (PPV), and negative predictive value (NPV)) are derived directly from quadrant counts, supplemented by the Hidden Burden Index, Facade Burden Index, and Facade-to-Hidden Ratio. The framework is illustrated with global pools of undiagnosed diabetes (~252 million) and hypertension (~580 million), substantial proportions of clinically labeled chronic obstructive pulmonary disease lacking spirometric confirmation (30%-60%), and the diagnostic shadow cast by depression, stroke, and other competing diagnoses.

The adapted Johari Window framework provides a structurally complete, operationalizable, and metric-yielding solution to limitations inherent in linear cascade models, enabling simultaneous assessment of underdiagnosis, overdiagnosis, diagnostic competition, and system-level diagnostic performance at the population scale.

## Introduction and background

Public health systems face a fundamental measurement challenge: how to account for the disease burden they cannot see and for diagnostic effort that fails to reach the disease that is in front of it. The paradox is stark. An estimated 252 million adults worldwide live with diabetes but remain unaware of their condition, representing 43% of total diabetes cases [1]. Simultaneously, 580 million people have undetected hypertension [2]. Yet within these same disease areas, substantial populations receive treatment for conditions they do not have. Between 30% and 60% of patients diagnosed with chronic obstructive pulmonary disease (COPD) lack spirometric confirmation of airflow limitation [3]. A further phenomenon, distinct from both underdiagnosis and overdiagnosis, is well documented: symptomatic individuals whose clinical encounters are captured by a competing label (for example, depression, stroke, or rheumatism), with the consequence that the target disease, although present, is never investigated. The coexistence of large-scale underdiagnosis, systematic overdiagnosis, and structured diagnostic competition is a population health reality that linear analytical frameworks do not jointly accommodate.

A care cascade is a sequential model that follows diagnosed individuals through an ordered stage, for example, diagnosis, treatment, and control, and quantifies attrition at each step. Since the formalization of the Rule of Halves in 1972 [4], cascade frameworks have been the dominant analytical structure for population gap analysis across major disease areas [4-11]. The Rule of Halves observed that half of people with hypertension are undetected, half of those detected are untreated, and half of those treated are uncontrolled, implying that only about one-eighth achieve control [4]. Hart generalized this logic in 1992, estimating that complete diagnosis and management of all chronic diseases would increase primary care workload by at least 12% [5].

The treatment cascade gained global prominence after Gardner and colleagues’ 2011 analysis of the HIV care continuum, which showed that only 30% of HIV-positive individuals achieved viral suppression [6]. The Joint United Nations Programme on HIV/AIDS subsequently adopted cascading targets of 90-90-90 by 2020 and, later, 95-95-95 by 2030, creating a global monitoring architecture [12,13]. This logic has since been extended to diabetes, tuberculosis, hepatitis C, mental health disorders, and virtually every major chronic disease area [7-11]. In India, the cascade has been applied both to tuberculosis, where only 39% of an estimated 2.7 million patients achieved one-year recurrence-free survival [11], and to mental health, where 70-92% of people with mental illness receive no formal treatment [14].

Despite widespread adoption, linear frameworks face mounting criticism. Powers and Miller identified the cascade’s inability to represent bidirectional movement, or 'churning,' that violates its unidirectional assumption [15], and Kay and colleagues documented that many patients skip stages entirely [16]. Silberzan and colleagues demonstrated in 52,434 French hypertensive adults that one-third of individuals with controlled blood pressure were structurally invisible to the cascade because they achieved control through non-standard pathways [17]. A separate limitation arises with competing diagnoses: when symptomatic individuals reach care, but the target disease is captured by an alternative label, the cascade classifies them as undetected when they are in fact misdetected. Our framework addresses the structural invisibility cascades exhibited, where individuals on non-standard pathways each occupy a defined quadrant at any cross-section. It does not, however, model the dynamics of movement between states; dynamic representation of inter-quadrant transitions would require a longitudinal multi-state extension, which we identify as future work (see section Limitations and Challenges of This Framework).

An asymmetry within epidemiology motivates this work. At the clinical scale, diagnostic performance is described by sensitivity, specificity, positive predictive value (PPV), and negative predictive value (NPV); standardized tools exist for reporting and risk-of-bias appraisal of every diagnostic instrument [18,19]. At the population scale, no analogous structural metrics are routinely reported. Effective coverage frameworks combine need, use, and quality into single proportions [20,21], but remain unidimensional and do not separate the four ways a health system can succeed or fail at population-level diagnosis.

The framework proposed here adapts the Johari Window, a 2×2 model developed by Joseph Luft and Harrington Ingham in 1955 for interpersonal awareness, cross-classifying what is known or unknown to ‘self’ against ‘others’ [22], to population-level gap analysis.

The adaptation is structural rather than metaphorical. Reframing the Luft-Ingham axes as true disease state and health-system labelling state yields a structure formally isomorphic to the 2×2 confusion matrix that already underpins clinical diagnostic evaluation, in which the reference standard is cross-classified against the test result to give true positives, false negatives, false positives, and true negatives. Our adaptation imports this established epidemiological object to the population scale, treating the disease axis as the reference and the system-labelling axis as the test. The framework, therefore, extends a tool epidemiology already uses rather than importing a psychological theory.

We contextualize the framework within the iceberg phenomenon of disease, which holds that clinically diagnosed cases represent only the visible tip of total disease burden [23]. To our knowledge, the Johari Window has not previously been adapted to population-level gap analysis. This manuscript formalizes the adaptation, derives its system-level diagnostic metrics, and illustrates them with a worked calculation.

## Review

Methods

Design

This study employed a narrative review design with conceptual framework development. A narrative review was chosen because the primary aim was conceptual synthesis and the derivation of an analytical structure rather than the pooled estimation of an effect, for which no common effect measure exists across the heterogeneous literature surveyed. Reporting follows the Scale for the Assessment of Narrative Review Articles (SANRA). We did not conduct a systematic review or meta-analysis, and we have deliberately not presented a PRISMA flow diagram or a meta-analytic summary, as both would misrepresent the methodology actually used; the disclosures below are intended to make the narrative approach transparent and repeatable on its own terms.

Information Sources and Search Strategy

We searched PubMed/Medical Literature Analysis and Retrieval System Online (MEDLINE) and Google Scholar for literature published between 2000 and January 2026, supplemented by backward and forward citation chasing of key papers and by landmark global burden sources outside that window, where they established foundational frameworks. Searching was purposive and iterative rather than exhaustive, consistent with a narrative synthesis.

Searches spanned five term clusters: (i) Johari Window applications in health and healthcare; (ii) population care cascades and the Rule of Halves; (iii) underdiagnosis and unmet need, including objective versus self-reported measures and screening and case-finding; (iv) overdiagnosis, overuse, and diagnostic stewardship; and (v) competing diagnoses, diagnostic overshadowing, and diagnostic substitution. For the first cluster, we combined ‘Johari Window’ with ‘healthcare’, ‘public health’, ‘epidemiology’, ‘disease surveillance’, ‘screening’, and ‘gap analysis’. For the second, we combined ‘Rule of Halves’, ‘treatment cascade’, ‘care cascade’, ‘continuum of care’, and ‘90-90-90’ with disease terms including ‘hypertension’, ‘diabetes’, ‘HIV’, ‘tuberculosis’, and ‘mental health’. For framework limitations, we searched ‘undiagnosed disease’, ‘diagnostic gaps’, ‘overdiagnosis’, ‘health system wastage’, and ‘cascade limitations’. For diagnostic competition, we searched ‘competing diagnoses’, ‘diagnostic overshadowing’, ‘diagnostic substitution’, and ‘attribution bias’ combined with ‘depression’, ‘stroke’, and ‘comorbidity’. We additionally consulted methodological literature on diagnostic-accuracy reporting and on estimation of test accuracy in the absence of a gold standard to ground the framework’s requirement for a defined disease criterion.

Selection and Synthesis

We prioritized, in descending order, systematic reviews and meta-analyses; large population-based studies published between 2000 and 2025; and seminal historical papers that established foundational frameworks. We excluded sources unrelated to framework structure, population coverage, documented diagnostic gaps, or empirical evidence of simultaneous underdiagnosis, overdiagnosis, or diagnostic competition. From the included sources, we extracted information on framework structure, population coverage, documented gaps, and quantitative estimates where available. We synthesized findings using thematic mapping to derive and justify the two-dimensional quadrant structure, the priority-ordered classification rule, the two facade subtypes, and the system-level diagnostic performance metrics. Source selection and synthesis were performed by the authors and reconciled by discussion; this is a narrative process and was not a duplicate independent screening as would be required for a systematic review.

Operational Axes

We adapted the Johari Window by redefining its axes for epidemiological application. The horizontal axis represents the true disease state (present or absent based on a defined clinical, biological, or laboratory criterion). The vertical axis represents health-system labeling state, with three operational levels: target-disease label (the system has assigned the diagnosis under study), competing label (the system has assigned a different diagnosis that occupies the clinical encounter without resolving the target condition), and no label (the system has no diagnostic record). The 2×3 cross-classification is collapsed to four mutually exclusive quadrants by the priority-ordered rule defined in the Results section.

Relationship to the Original Johari Window

The original Johari Window (Luft and Ingham, 1955) is a heuristic for interpersonal awareness that cross-classifies information about an individual by what is known or unknown to the self and to others, yielding four regions: Open (known to self and others), Blind spot (known to others, not the self), Hidden (known to the self, not others; termed the "Facade" in some expositions), and Unknown (known to neither) [22]. Our adaptation preserves the 2×2 form but respecifies both axes for population-level application: the "self" axis becomes the true disease state (present or absent by a gold-standard criterion), and the "others" axis becomes the health-system labeling state. Under this re-axing, the cells map positionally, Open, Hidden, and Unknown, retain their names with directly analogous meaning, while the cell the original calls the Blind Spot is renamed the Facade and given new content (a system label without an actionable target diagnosis). Two further departures distinguish the adaptation: the labeling axis is expanded from the original binary to three operational levels (target label, competing label, and no label) and collapsed to four quadrants by the priority-ordered rule; and the Facade quadrant is decomposed into two co-equal subtypes, Type I (diagnostic misclassification) and Type II (diagnostic competition), neither of which has an analogue in the interpersonal model. These departures are summarized in Table 1. Note that "Facade" in the original is a synonym for the Hidden quadrant; in this adaptation, the term is reassigned to the cell the original calls the Blind Spot.

**Table 1:** Relationship between the original Johari Window and the adapted framework. "Facade" in the original model is a synonym for the Hidden quadrant; the four canonical regions are Open, Blind Spot, Hidden, and Unknown.

Element	Original Johari Window (Luft & Ingham, 1955)	Adapted framework (this paper)	Nature of the change
Domain/unit of analysis	Interpersonal and group awareness: the individual	Population diagnostic status: the health system	Re-contextualisation
Horizontal axis	Known/unknown to self	True disease state: present/absent by gold-standard criterion	Axis re-specified (subjective awareness → objective biological reality)
Vertical axis	Known/unknown to others (binary)	Health-system labelling state: target label / competing label / no label	Axis re-specified and expanded from two to three levels
Open	Known to self and others	Disease present + target-disease label	Name retained; meaning analogous
Blind Spot	Known to others, not to self	Renamed Facade, system label without an actionable target diagnosis	Renamed and repurposed
Hidden (a.k.a. Facade)	Known to self, not to others	Disease present + no label	Name retained; meaning analogous; "Facade" not used in this sense here
Unknown	Known to neither	Disease absent + no label	Name retained; meaning analogous
Facade subtypes	None	Type I (misclassification); Type II (diagnostic competition): co-equal	New; no analogue in the original
Purpose/output	Enlarge the Open area via disclosure and feedback	Quantify quadrant counts; derive system sensitivity, specificity, positive predictive value, negative predictive value, hidden burden index, false burden index, and false harmony ratio.	New analytical function

Results

The Adapted Johari Window Framework

**Figure 1:**
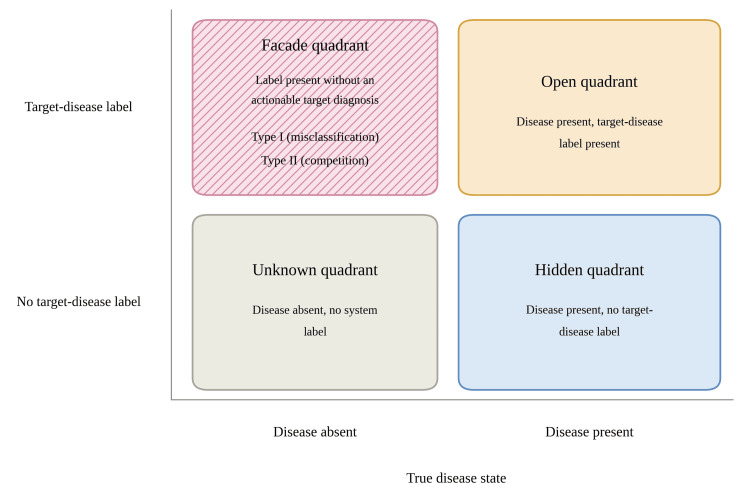
The adapted Johari Window framework for population gap analysis. The horizontal axis represents the true disease state (present/absent by a gold-standard criterion); the vertical axis represents the health-system labeling state. Each individual occupies one quadrant: Open (disease present, target-disease label), Hidden (disease present, no label), Unknown (disease absent, no label), and Facade (a system label without an actionable target diagnosis). The Facade quadrant comprises two co-equal subtypes assigned by the priority-ordered rule (Figure 2): Type I (diagnostic misclassification: disease absent with a target-disease label) and Type II (diagnostic competition: disease present with a competing label); the quadrant accordingly spans both disease states. Created using the matplotlib package of Python (Matplotlib Development Team, San Francisco, CA, USA).

The framework generates four mutually exclusive, jointly exhaustive population quadrants, each representing a specific combination of true disease state and health-system labeling state. It is operationalized through two binary indices and a priority-ordered classification rule (Figure 1).

Index A, true disease state: An individual is disease-positive if they meet a pre-specified gold-standard criterion appropriate to the condition (for example, spirometric airflow limitation for COPD; sustained blood pressure ≥140/90 mmHg, or ≥130/80 mmHg by guideline, for hypertension; fasting glucose ≥126 mg/dL or HbA1c ≥6.5% for diabetes; or bacteriologically confirmed *Mycobacterium tuberculosis* for active tuberculosis). Where a single gold standard is unavailable in survey-based applications, a composite operational definition may be substituted, with sensitivity analyses across alternative definitions. We return to this important constraint in the section on Limitations and Challenges of This Framework.

Index B, health-system labeling state: An individual occupies one of three labeling states: (i) target-disease label (registry entry, physician diagnosis of the target condition, or programmatic notification); (ii) competing label (a non-target diagnosis that occupies the diagnostic encounter, within-domain, such as rheumatism instead of osteoarthritis, or across-domain, such as depression or stroke obscuring osteoarthritis-related functional decline); and (iii) no label.

*Priority-Ordered Classification Rule* 

**Figure 2:**
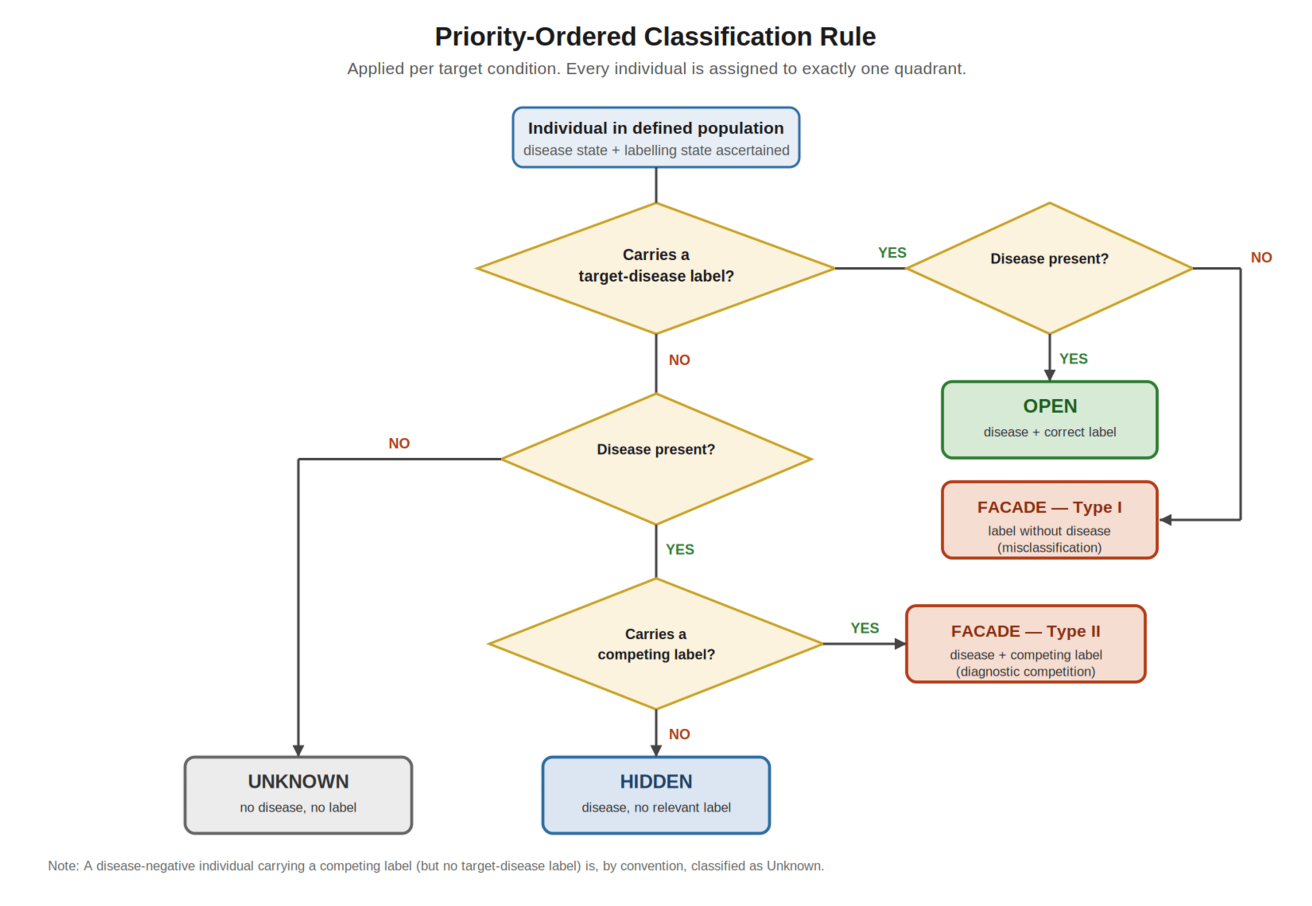
The priority-ordered classification rule. Applied per target condition, the rule assigns every individual to exactly one quadrant. The Facade quadrant accommodates both Type I (label without disease) and Type II (disease with a competing label).

The 2×3 cross-classification of disease state and labeling state contains six logical cells. We collapse these to four quadrants by the following priority-ordered rule (Figure 2), which ensures that every individual occupies exactly one quadrant for the condition under study.

Open: any individual with a target-disease label, on the principle that a gold-standard-confirmed diagnosis persists in the record irrespective of current symptomatology.

Facade: any disease-negative individual carrying a target-disease label (Type I, diagnostic misclassification) or any disease-positive individual carrying a competing label without a target-disease label (Type II, diagnostic competition).

Hidden: any disease-positive individual with no target-disease label and no competing label (the residual undiagnosed).

Unknown: any disease-negative individual with no target-disease label and no competing label. A disease-negative individual carrying a competing label is, by convention, classified as Unknown; this minor edge case is rare in practice and does not materially affect the framework’s utility.

The Four Quadrants

Open: Individuals with disease who carry the target-disease label; this is the exclusive focus of the Rule of Halves and treatment cascades. Current estimates place approximately 337 million people with diagnosed diabetes and roughly 700 million with diagnosed hypertension in this quadrant [1,2]. Visibility under the correct disease label is what makes evidence-based management possible.

Hidden: True disease is present, but there is no target-disease label and no competing label. It comprises preclinical disease (incubation or latent period); subclinical, detectable disease accessible only through screening or case-finding; and symptomatic disease that remains undiagnosed owing to non-presentation, missed diagnosis, misclassification, or access barriers. The principal lever is secondary prevention. The International Diabetes Federation (IDF) places 252 million adults with undiagnosed diabetes [1]; the Noncommunicable Disease Risk Factor Collaboration (NCD-RisC) indicates approximately 580 million adults with undetected hypertension [2]; the World Health Organization (WHO) 2025 Global Tuberculosis Report estimated 2.4 million incident tuberculosis cases never diagnosed or reported [24]; and approximately 13% of people living with HIV remain undiagnosed [25].

Facade: A system label without an actionable target diagnosis. The original framing, confined to false-positive labelling, was structurally incomplete. We distinguish two mechanistically distinct subtypes.

Type I, diagnostic misclassification (label without disease): Disease-negative individuals carrying a target-disease label arise from false-positive tests, overdiagnosis of indolent abnormalities, absent confirmatory testing, or inappropriately broad thresholds. Diab and colleagues documented that 30%-60% of patients with physician-diagnosed COPD do not meet spirometric criteria [3]; Bell and colleagues showed that the 2017 American College of Cardiology (ACC)/American Heart Association (AHA) guidelines could misclassify 38% of normotensive individuals on single-visit measurement [26]; Welch and Black estimated that approximately 25% of mammographically detected breast cancers and 60% of prostate-specific antigen (PSA)-detected prostate cancers represent overdiagnosis [27]. The principal lever is diagnostic stewardship.

Type II, diagnostic competition (label without resolution of the target disease): Disease-positive individuals carrying a competing (non-target) label that has captured the diagnostic encounter. The label is correct for what it labels; the failure is that it absorbs attention that the target disease should have triggered. This phenomenon is documented in the diagnostic-overshadowing literature, in which physical symptoms in people with mental illness are misattributed to the psychiatric diagnosis, with consequent underdiagnosis and undertreatment of physical disease [28-30]. Within-domain examples include rheumatism or osteoporosis labels among individuals with osteoarthritis; across-domain examples include depression labels among individuals whose joint pain is attributed to mood disorders and stroke labels that absorb post-stroke mobility limitations without separate musculoskeletal evaluation. The principal lever is reallocation of diagnostic attention, are structured screening for the target disease embedded in the settings where competing-label patients are already present. We note that the specific across-domain examples are best documented qualitatively; we present them as illustrative of the Type II mechanism rather than as quantified estimates and identify their formal quantification as a research priority.

The two subtypes have non-overlapping policy implications: Type I expands when test specificity is poor or confirmatory testing is omitted (response: tighten the diagnostic process), whereas Type II expands when comorbidity and attention structures cause one diagnosis to absorb another’s encounter (response: redistribute screening into existing care contacts). A health system may exhibit a small Type I and a large Type II Facade, or the reverse, and the appropriate intervention differs accordingly.

Unknown: Disease-negative individuals with no system label, comprising a not-at-risk stratum (no exposure to relevant risk factors; the denominator that makes screening expensive in low-prevalence settings) and an at-risk stratum (exposed but not yet diseased; the principal target for primary prevention). In tuberculosis contact tracing, Alsdurf and colleagues found that fewer than 5% of individuals with latent infections are diagnosed and treated, leaving the majority distributed across the Unknown and Hidden quadrants [31].

Population-Level Diagnostic Performance Metrics

**Figure 3:**
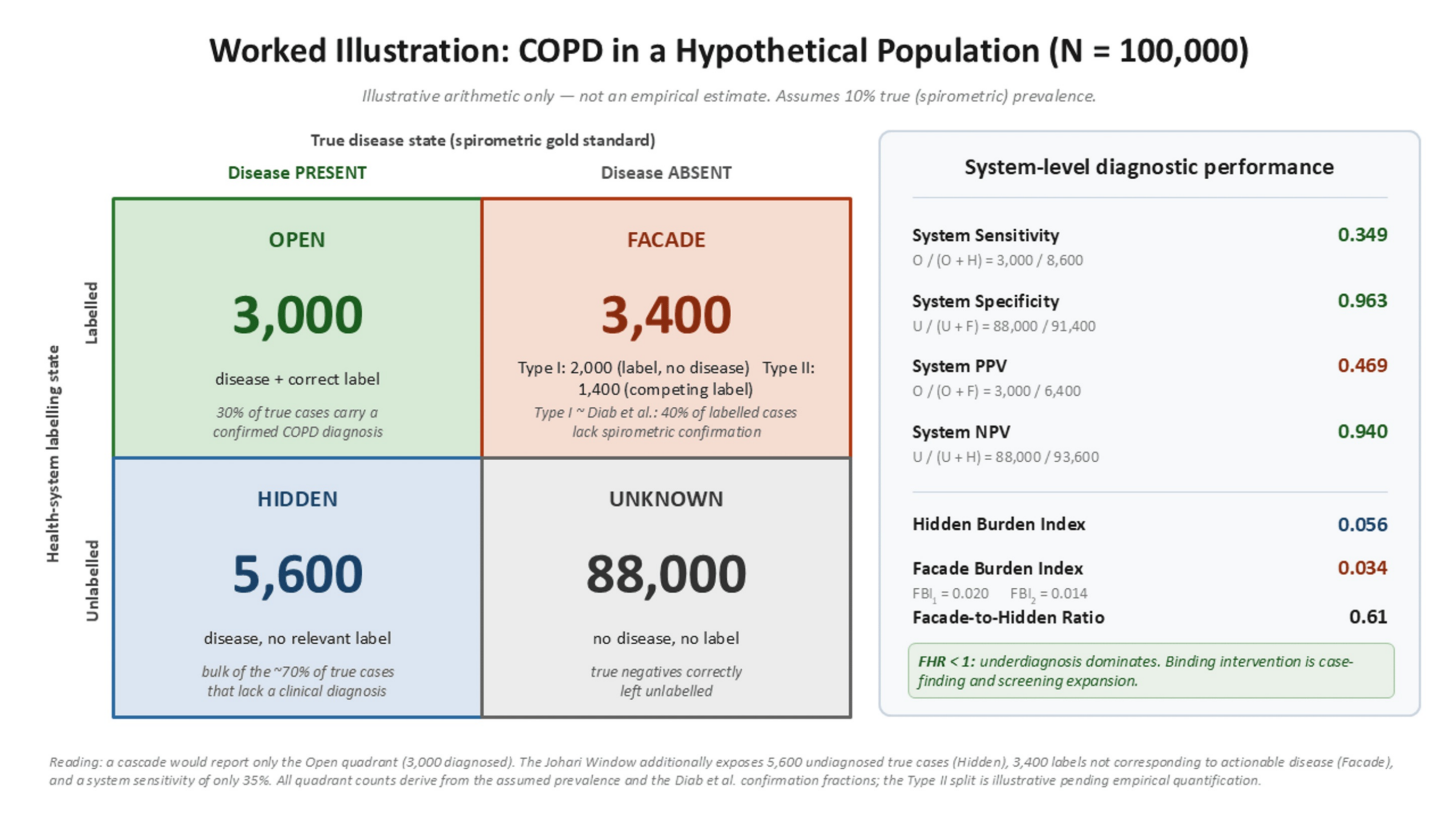
Worked illustration of the framework for COPD in a hypothetical population (N = 100,000), showing quadrant counts and the derived system-level metrics. Illustrative arithmetic only, not an empirical estimate. COPD: chronic obstructive pulmonary disease; PPV: positive predictive value; NPV: negative predictive value

The four-quadrant structure is formally isomorphic to a 2×2 confusion matrix at the population scale. Treating the disease axis as the reference (true positive versus true negative) and the system-labelling axis as the test (label-positive versus label-negative) yields four system-level diagnostic performance metrics that translate well-established clinical concepts to the health-system scale. Throughout, O, H, F, and U denote the population counts in the Open, Hidden, Facade, and Unknown quadrants, with N = O + H + F + U. Table 2, which presents these metrics and is, with the worked illustration in Figure 3, among the more directly usable parts of the framework, summarises the four primary metrics, three derived burden indices, and their policy interpretation. Table 2 summarizes the four primary metrics, three derived burden indices, and their policy interpretation.

**Table 2:** System-level diagnostic performance metrics derived from Johari Window quadrant counts. Note: O, H, F, and U denote Open, Hidden, Facade, and Unknown quadrant populations; N is the total population. PPV: positive predictive value; NPV: negative predictive value

Metric	Formula	Interpretation	Policy lever when degraded
System Sensitivity (Sens_sys_)	O / (O + H)	Proportion of all true cases correctly identified by the health system	Expand active case-finding; widen screening coverage; reduce access barriers
System Specificity (Spec_sys_)	U / (U + F)	Proportion of all true non-cases correctly left unlabelled	Diagnostic stewardship; mandate confirmatory testing; address Type I and Type II Facade
System PPV (PPV_sys_)	O / (O + F)	Probability that an individual carrying any system label genuinely has the target disease	Tighten diagnostic confirmation; reduce competing-label attribution bias
System NPV (NPV_sys_)	U / (U + H)	Probability that an unlabelled individual is genuinely disease-free	Improve case-finding among the unlabelled; raise screening coverage in at-risk strata
Hidden Burden Index (HBI)	H / N	Population prevalence of true disease that is unlabelled by the system	Targets case-finding and screening expansion at the population level
Facade Burden Index (FBI)	F / N	Population prevalence of system labels that do not correspond to actionable target disease (Type I + Type II combined)	Diagnostic stewardship and reallocation of clinical attention
Facade-to-Hidden Ratio (FHR)	F / H	Balance between overdiagnostic activity and undiagnostic failure. FHR <1: underdiagnosis dominates. FHR >1: overdiagnosis or diagnostic competition dominates	Indicates whether the binding intervention is screening expansion (FHR <1) or stewardship and reallocation (FHR >1)

The four properties deserve emphasis. First, prevalence dependence: system sensitivity and specificity are intrinsic properties of the system’s case-finding and labelling discipline, whereas system PPV and NPV are jointly governed by these and by underlying prevalence - in low-prevalence settings, PPV will be low even when Specificity is high, the population-level analogue of the Bayesian screening paradox. Second, structural completeness: the four metrics jointly partition the population, making trade-offs visible in a way single-proportion metrics cannot. Third, monitoring tractability: a screening expansion that maintains specificity should raise system sensitivity and system NPV without depressing system PPV; an expansion that sacrifices specificity raises system sensitivity but inflates the Facade Burden Index and depresses system PPV, signalling diagnostic stewardship as the next intervention. Fourth, granularity: where data permit, the Facade can be partitioned into Type I and Type II contributions $FBI_1 = \frac{F_1}{N},\; FBI_2 = \frac{F_2}{N}$, directly informing the appropriate intervention.

A Worked Illustration

To demonstrate that the metrics are computable and to make the framework concrete, Figure 3 presents a worked illustration for COPD in a hypothetical population of 100,000 adults. This is an illustrative calculation of the metric arithmetic, not an empirical estimate. Assuming a true (spirometric) prevalence of 10% and applying the confirmation fractions reported by Diab and colleagues, approximately 70% of true cases lack a clinical diagnosis, and approximately 40% of labeled cases lack spirometric confirmation [3]. The four quadrants are populated as shown. The resulting system sensitivity of 0.35 and system specificity of 0.96, with a facade-to-hidden ratio of 0.61, illustrate a system in which underdiagnosis dominates: a cascade restricted to the Open quadrant would report only the 3,000 diagnosed cases and their internal treatment metrics, while the Johari Window additionally exposes 5,600 undiagnosed true cases and 3,400 labels not corresponding to actionable disease. All quadrant counts are derived from the assumed prevalence and the cited confirmation fractions; they illustrate the arithmetic, and the Type II split is presented as illustrative pending empirical quantification.

Comparison with Linear Frameworks

The critical distinction lies in structural completeness. The Rule of Halves and treatment cascades operate exclusively within the Open quadrant. The cascade denominator - total disease prevalence - includes the Hidden quadrant as an estimated but unmeasured population. The Facade quadrant is invisible to cascade logic, which assumes all individuals carrying the target-disease label truly have the target condition; diagnostic competition is invisible a fortiori, since cascades have no place for individuals carrying a non-target label. Table 3 presents population estimates across the four quadrants for selected conditions, distinguishing directly measured surveillance estimates from illustrative or inferential entries.

**Table 3:** Population distribution across Johari Window quadrants for selected conditions. Open and Hidden values are directly measured surveillance estimates from the cited sources. Facade entries are marked as measured where a verification study exists (hypertension Type I, COPD Type I) and as illustrative otherwise; Type II (diagnostic competition) is not yet systematically quantified for any condition. COPD: chronic obstructive pulmonary disease; BP: blood pressure; HbA1c: haemoglobin A1c.

Condition	Open (Diagnosed, true disease)	Hidden (Undiagnosed, true disease)	Facade (Type I + Type II)	Source
Diabetes (Global, 2024)	337 million	252 million (43% of total)	Type I: false positives from HbA1c in hemoglobinopathies; Type II: under-investigated	IDF Atlas 2025 [1]
Hypertension (Global, 2019)	~700 million	~580 million (46% of total)	Type I: 38% misclassified by single-visit BP [21]; Type II: variable	NCD-RisC 2021 [2]
COPD (Global)	70 million (30% of true cases)	160 million (70% of true cases)	Type I: 30–60% of clinical diagnoses lack spirometric confirmation [3]	Diab et al. 2018 [3]
HIV (Global, 2025)	35.4 million	5.3 million (13% of total)	Type I: minimal (high test specificity)	UNAIDS 2025 [20]
Tuberculosis (Incident cases, 2025)	8.1 million notified	2.4 million missing cases	Type I: not systematically quantified; Type II: respiratory symptoms attributed to COPD/asthma	WHO TB Report 2025 [19]

Empirical Evidence for All Four Quadrants

Several studies have inadvertently documented all four quadrants, though without a unifying framework. Silberzan and colleagues identified pathways corresponding to each quadrant in hypertension, and their finding that one-third of controlled patients followed non-standard pathways invisible to cascade models directly demonstrates the inadequacy of linear frameworks [17]. Holm and colleagues separated diabetes prevalence into known (Open) and screen-detected (Hidden) cases, finding 74% previously diagnosed [32]; subsequent work has documented substantial false-positive rates with HbA1c testing in haemoglobinopathies and kidney disease [33]. The coexistence of underdiagnosis and overdiagnosis in COPD provides the clearest demonstration: approximately 70% of spirometry-confirmed cases lack a clinical diagnosis (Hidden), while 30%-60% of physician-diagnosed cases lack confirmation (Facade Type I) [3]. Type II Facade, diagnostic competition, has been described qualitatively but rarely quantified; it is best documented in the diagnostic-overshadowing literature, where physical symptoms in people with mental illness are misattributed to the psychiatric diagnosis [28-30]. In any population in which competing-label morbidity is common, the two subtypes may differ substantially in magnitude, and an analysis that collapses them risks misdirecting policy toward stewardship when the binding problem is reallocation, or vice versa.

Discussion

Principal Findings

This paper proposes an adaptation of the Johari Window framework to population-level public health gap analysis. By reframing the original awareness axes as true disease state and health-system labelling state, applying a priority-ordered classification rule, and distinguishing two subtypes of the Facade quadrant, we generate a structurally explicit classification that captures all jointly meaningful combinations of disease and labelling. Contextualised within the iceberg phenomenon, the four quadrants, supplemented by formal system-level diagnostic metrics and the worked illustration in Figure 2, provide both intuitive representations and quantitatively tractable measures of populations that linear cascade models do not represent.

Current cascade-based gap analysis focuses on the Open quadrant. It estimates but does not structurally represent the Hidden quadrant; it has no place for the Facade quadrant; and it treats the Unknown quadrant as background. We emphasize that linear cascades were designed to quantify attrition among diagnosed cases, not to detect overdiagnosis or competing diagnoses; the limitation we identify is one of scope, not of internal validity. The literature does, separately, address each phenomenon; overdiagnosis, in particular, is extensively documented [27,34], and we do not claim otherwise; our argument is that comprehensive gap analysis now requires a single structure that encompasses what the cascade was never intended to capture. The scale is substantial: 252 million people with undiagnosed diabetes and 580 million with undetected hypertension in the Hidden quadrant [1,2], alongside millions in the Facade quadrant receiving unnecessary labels (Type I) or whose encounters are absorbed by competing labels (Type II).

Strengths and Novel Contributions

The framework offers four advantages over unidimensional cascade models. First, structural completeness: applied to a single target condition, the priority-ordered rule assigns every individual to exactly one quadrant. Multimorbid individuals are classified once per condition and may legitimately occupy different quadrants for different diseases, so the framework accommodates multimorbidity through parallel disease-specific application rather than a single global assignment; individuals whose disease status is genuinely indeterminate are handled by the gold-standard rule and its sensitivity analyses (see Limitations and Challenges of This Framework). Second, simultaneous visibility of underdiagnosis, overdiagnosis, and diagnostic competition, with the trade-offs between them made explicit: expanding screening to reduce the Hidden quadrant increases the Type I Facade unless specificity is perfect, while reducing the Type II Facade requires investment in clinical settings that the screening program does not reach. Third, the formal derivation of system-level diagnostic metrics computable directly from quadrant counts, as demonstrated in the worked illustration. Fourth, the operationalization of diagnostic competition through the Type I/Type II decomposition, which translates a long-standing qualitative observation into a tractable category with its own metric and policy lever.

Limitations and Challenges of This Framework

The framework faces several challenges, and we have not validated it empirically; the contribution is conceptual and structural. First, population classification requires a gold-standard criterion for true disease, yet such criteria may be ambiguous, evolving, or threshold-dependent, and for several chronic diseases, a single gold standard does not exist; hypertension and diabetes thresholds have changed over time and differ between guidelines [26,33]. This is the framework’s most consequential constraint: where disease status is uncertain, quadrant assignment requires a pre-specified tie-breaking rule, and sensitivity analyses across alternative definitions and estimation may need to draw on latent-class methods developed precisely for the absence of a gold standard [35,36]. Second, data requirements are substantial: complete ascertainment of all four quadrants is not directly feasible in most routine surveillance systems, which lack verification data on labeled cases and competing-label data. Approximate quadrant estimation is nonetheless achievable where population screening and diagnosed-prevalence data coexist, as in large household and aging surveys, including the National Family Health Survey (NFHS), the Longitudinal Ageing Study in India (LASI), the National Health and Nutrition Examination Survey (NHANES), the Health and Retirement Study (HRS), and the English Longitudinal Study of Ageing (ELSA). We identify the development of routine verification substudies as a methodological priority, recognizing that this burden is greatest in low-resource settings where the burden of NCDs is highest. Third, the framework treats disease and labeling states as discrete categories, sacrificing some nuance for tractability. Fourth, it provides cross-sectional snapshots rather than dynamic representations of movement between quadrants; longitudinal adaptation would require Markov-style transition structures, which lie beyond the present scope.

Implications for Research, Practice, and Policy

For surveillance design, the framework allows health systems to set explicit targets for system sensitivity (case-finding) while monitoring system specificity (label discipline) and to decompose the Facade into Type I and Type II contributions to guide between-strategy choice. For resource allocation, interventions can be evaluated across all four quadrants and both Facade subtypes rather than against a single coverage proportion: community-based hypertension screening may reduce the Hidden quadrant at the cost of expanding the Type I Facade through white-coat hypertension, while integrating brief musculoskeletal screening into mental health services may reduce the Type II Facade with limited effect on the Hidden quadrant. For NCD programmes, the WHO Global Action Plan for NCDs and the Sustainable Development Goal (SDG) 3.4 indicators are specified as coverage targets and do not include indicators for diagnostic accuracy, overdiagnosis, or diagnostic competition [37]; the NCD Countdown 2030 Collaborators found that only 19% of countries for women and 16% for men were on track for SDG target 3.4, with inadequate detection a primary barrier [38]. A four-quadrant assessment with system-level metrics would supply a more discriminating account of where detection is failing. We note that diagnostic errors are themselves common. Singh and colleagues estimated that approximately 12 million US adults are affected annually in outpatient care, defined as missed opportunities to make a timely or correct diagnosis based on available evidence [39], and that the framework provides a population-level accounting structure for these error types.

Future Research Directions

Empirical validation studies should apply the framework, with formal calculation of system-level metrics, to disease areas with available gold-standard data; COPD is a natural starting point for Type I Facade quantification and conditions with well-documented competing-label comorbidity (musculoskeletal disease in aging populations, physical disease in psychiatric populations) for Type II. Methodological development should extend the framework to disease-severity gradations and dynamic transitions. Economic evaluations should compare the marginal cost per true case detected (reducing Hidden), per Type I false positive avoided (improving specificity), and per Type II case recovered (integrating target-disease screening into competing-label care contexts). Cross-national comparisons could identify systems achieving a favorable balance across all four quadrants and both Facade subtypes, comparisons single-cascade analyses cannot perform.

## Conclusions

Linear cascade frameworks have dominated public health gap analysis for five decades because they offer intuitive, tractable methods for quantifying deficits among diagnosed populations. Their unidimensional structure, however, leaves three population segments structurally invisible to the cascade itself: undiagnosed disease enters only as an external denominator estimate rather than a represented compartment; populations carrying labels without actionable disease have no place in cascade logic; and individuals whose target disease is captured by a competing label are not distinguished from the undiagnosed. None of this implies these phenomena are absent from the wider literature. Overdiagnosis in particular is extensively documented, but no single linear framework renders them jointly analyzable.

The adapted Johari Window provides a structurally explicit alternative. Its four quadrants, Open, Hidden, Facade (partitioned into diagnostic misclassification and diagnostic competition), and Unknown, jointly partition the population, and the system-level metrics derived from quadrant counts express population diagnostic performance in the same terms used at the clinical scale. We have demonstrated that these metrics are computable through a worked illustration while emphasizing that empirical validation in applied datasets is the necessary next step. The populations under-represented by linear cascades (those with undiagnosed disease in the Hidden quadrant, those carrying unnecessary labels, and those whose target disease is absorbed by competing diagnoses) are too large and consequential to leave outside the analytical frame.
